# Hyperammonemia Is Associated with Increasing Severity of Both Liver Cirrhosis and Hepatic Encephalopathy 

**DOI:** 10.1155/2016/6741754

**Published:** 2016-10-25

**Authors:** Abidullah Khan, Maimoona Ayub, Wazir Mohammad Khan

**Affiliations:** KTH Peshawar, Peshawar, Pakistan

## Abstract

*Background*. Hyperammonemia resulting from chronic liver disease (CLD) can potentially challenge and damage any organ system of the body, particularly the brain. However, there is still some controversy regarding the diagnostic or prognostic values of serum ammonia in patients with over hepatic encephalopathy, especially in the setting of acute-on-chronic or chronic liver failure. Moreover, the association of serum ammonia with worsening Child-Pugh grade of liver cirrhosis has not been studied.* Objective*. This study was conducted to solve the controversy regarding the association between hyperammonemia and cirrhosis, especially hepatic encephalopathy in chronically failed liver.* Material and Methods*. In this study, 171 cirrhotic patients had their serum ammonia measured and analyzed by SPSS version 16. Chi-squared test and one-way ANOVA were applied.* Results*. The study had 110 male and 61 female participants. The mean age of all the participants in years was 42.33 ± 7.60. The mean duration (years) of CLD was 10.15 ± 3.53 while the mean Child-Pugh (CP) score was 8.84 ± 3.30. Chronic viral hepatitis alone was responsible for 71.3% of the cases. Moreover, 86.5% of participants had hepatic encephalopathy (HE). The frequency of hyperammonemia was 67.3%, more frequent in males (*N* = 81, *z*-score = 2.4, and *P* < 0.05) than in females (*N* = 34, *z*-score = 2.4, and *P* < 0.05), and had a statistically significant relationship with increasing CP grade of cirrhosis (*χ* ^2^(2) = 27.46, *P* < 0.001, Phi = 0.40, and *P* < 0.001). Furthermore, serum ammonia level was higher in patients with hepatic encephalopathy than in those without it; *P* < 0.001.* Conclusion*. Hyperammonemia is associated with both increasing Child-Pugh grade of liver cirrhosis and hepatic encephalopathy.

## 1. Introduction

Chronic liver disease (CLD) and liver cirrhosis are clinicopathologically defined disease entities. The main causes of cirrhosis of the liver, include chronic infection by viral agents (hepatitis B and C viruses), as well as metabolic toxic/drug-induced and autoimmune causes, resulting in persistent inflammation and progressive fibrosis. In fact, it is the chronic activation of the wound healing response which is the major driving force for progressive accumulation of extracellular matrix (ECM) components, eventually leading to liver cirrhosis and hepatic failure [1].

The symptoms associated with chronic liver disease depend on the level of degeneration within the liver itself. The early stages are often asymptomatic and can only be detected by specific medical tests like liver function tests and abdominal ultrasound. However, liver diseases which have progressed to a chronic stage can be recognized by mental confusion, severe jaundice, coagulopathy, and so forth [2].

Normal blood ammonia concentration is less than 35 *μ*mol/L. Normally, blood ammonia comes from the bacterial hydrolysis of urea and other nitrogenous compounds in the intestine, the purine-nucleotide cycle and amino acid transamination in skeletal muscle, and other metabolic processes in the kidneys and liver [3, 4].

Mathews suggested an association between hyperammonemia and confusion in 1922 [5]. Similarly, further studies demonstrated a role of hyperammonemia, in the causation of hepatic coma [6, 7]. However, hyperammonemia alone is not always responsible for causing hepatic encephalopathy and, at times, patients with hepatic coma may have normal blood ammonia levels and vice versa [8–10].

The rationale of this study was to document an association between hyperammonemia and hepatic encephalopathy and to correlate serum ammonia with worsening Child-Pugh score/grade.

## 2. Material and Methods

This descriptive, cross-sectional study was conducted in the department of medicine of Khyber Teaching Hospital, Peshawar, Pakistan, between February and August 2016. A total of 250 patients were assessed for their suitability in the study. However, only 171 patients satisfied the inclusion and exclusion criteria and were included in the final study.

The inclusion criteria included (1) all patients with established chronic liver disease (CLD) with or without hepatic encephalopathy (CLD was diagnosed on the basis of any of the clinical features like finger clubbing, palmar erythema, spider naevi, splenomegaly, hepatomegaly or shrunken liver, or the persistent elevation for more than 6 months of the liver enzymes, namely, alanine aminotransferase (ALT), aspartate aminotransferase (AST), alkaline phosphatase (ALP), and gamma glutamyl transferase (GGT), plus a positive abdominal ultrasound for irregular liver margins, coarse liver appearance, and a dilated portal vein measuring 13–15 mm or more), (2) both genders, and (3) patients aged from 18 to 70 years.

The exclusion criteria included (1) patients already diagnosed with inherited urea cycle defect, (2) patients with chronic kidney disease, (3) hypertensive patients, (4) patients on parental nutrition or high protein diet, and (5) patients on drugs like alcohol, barbiturates, diuretics, valproate, and narcotics and smokers.

This study was approved by the hospital ethics review committee. An informed written consent was obtained from every participant. All the data was recorded on a structured questionnaire, including demographic details, duration of CLD, and serum ammonia levels. After 6 hours of overnight fasting, three mL of venous blood was drawn from every participant. Serum ammonia was determined by the same technician in the laboratory of our hospital. Those who had serum ammonia levels greater than 35 umol/L were classified as having hyperammonemia.

Data was stored and analyzed by the statistical program, SPSS Version 16. All the quantitative variables like age, blood ammonia levels, disease duration, and so forth were analyzed for mean ± standard deviation. Frequencies and percentages were calculated for qualitative variables like gender, hyperammonemia, Child-Pugh (CP) grade of liver cirrhosis, and so forth. By using Chi-square test, hyperammonemia was stratified amongst age, gender, disease severity (CP grade), grades of hepatic encephalopathy (HE grade), and so forth to see effect modification. To see any evidence of relationship of serum ammonia with both CP and HE grades, one-way ANOVA was run. *P* value of less than 0.05 was taken as criterion standard.

## 3. Results

The mean age of all the participants in years was 42.33 ± 7.60. The minimum age was 26 and the maximum was 54 years. The mean duration of chronic liver disease was 10.15 years (SD = 3.53). The study group comprised 110 (64.3%) males and 61 females (35.7%). 75% of the patients were Pakistanis (*N* = 128), against 25% of Afghanis (*N* = 43).

The most common cause of chronic liver disease was chronic viral hepatitis either chronic hepatitis B or C or both. Metabolic causes of the chronic liver disease, namely, Wilson's disease, hemochromatosis, and alpha-1 antitrypsin deficiency, and so forth, were the least encountered etiologies (Table 1). The mean Child-Pugh score was 8.84 ± 3.30 (Table 2).

**Figure 1:**
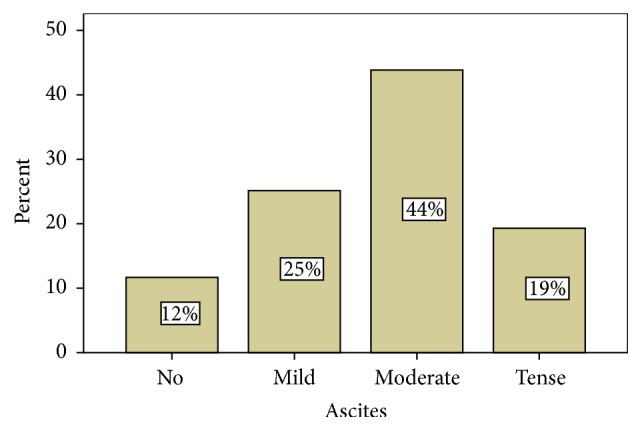
Percentage of patients with different grades of ascites on ultrasonography.

All the patients had splenomegaly. The splenic size was measured in centimeters on ultrasound of the abdomen (M = 16.57 and SD = 1.86%). It is worth mentioning that 32% of the patients had hematemesis which brought them to the hospital against 68% who had no such evidence. Ascites was another problem reported by most of our patients (Figure 1). Other clinical features of chronic liver disease, in our patients, included palmar erythema (45%), leuckonychia (50%), spider naevi (33%), jaundice (86%), cachexia (15%), purpura and ecchymosis (49%), fetor hepaticus (7%), and flapping tremor (43%). Liver disease related complications were commonly seen as well (Table 3). The ultrasonography findings are shown as follows (Table 4).

Amongst all the participants, 86.5% had hepatic encephalopathy (HE). West-Haven classification was used to stratify patients with HE into different groups (Table 5). The mean serum ammonia level was 45.91 ± 16.97. The minimum value of blood ammonia was 25 *μ*mol/L against the maximum value of 99 *μ*mol/L. 67.3% of the participants had hyperammonemia. The association of hyperammonemia with different grade of HE is given below (Table 6).

In order to see the relationship between serum ammonia and hepatic encephalopathy, one-way ANOVA was used. The assumption of homogeneity of variance was tested which showed violation and Levene's *P* < 0.05. However, robust tests of equality of means (Welch and Brown-Forsythe) were statistically significant *P* < 0.001. The results of ANOVA showed that serum ammonia level was higher in patients with high-grade HE, that is, grades 3 and 4 (*N* = 40, M = 51.98 ± 8.01, and *P* < 0.001 and *N* = 29, M = 76.28 ± 10.70, and *P* < 0.001, resp.), than in the patients with low-grade HE, that is, grades 1 and 2 (*N* = 41, M = 35.17 ± 4.72, and *N* = 38.  M = 37.61 ± 4.22, resp.). Patients with no evidence of HE had the lowest levels of blood ammonia (*N* = 23, M = 29.91 ± 3.32, and *P* < 0.001). Thus one-way ANOVA showed a statistically significant difference between the groups with regard to serum ammonia level; *F*(4) = 229.29, *P* < 0.001, and Eta squared = 0.85. Hence, it can be postulated, that the higher the grade of hepatic coma, the higher the blood ammonia level will be.

It must be noted that post hoc analysis via Tukey HSD revealed that patients with grades 3 and 4 of HE were significantly different from patients with either no coma or grade 1 or 2 coma; *P* < 0.001. However, there was no statistically significant difference between patients with grade 1 coma and those with grade 2; *P* = 0.49. There was little but statistically significant difference between patients with grade 1 or grade 2 coma and patients with no coma; *P* < 0.02 and *P* < 0.001, respectively.

Hyperammonemia was also stratified amongst gender using Chi-square test. The results showed that hyperammonemia was more prevalent in males (*N* = 81 and *z*-score = 2.4) than in females (*N* = 34 and *z*-score = 2.4) at a statistically significant level (*χ* ^2^(1) = 5.70 and *P* = 0.01). The effect size was again significant; Phi = 0.18 and *P* = 0.01.

Finally *χ* ^2^ test was used to assess any association between hyperammonemia and the different grades of cirrhosis taking *P* < 0.005 as criterion standard. The results revealed that 46.1% of patients (*N* = 53) with Child-Pugh grade C cirrhosis had hyperammonemia at statistically significant level (*z*-score = 4.3 and *P* < 0.05). In contrast, 50% (*N* = 28) of the patients with grade A cirrhosis had no hyperammonemia (*z*-score = 4.6 and *P* < 0.05). In patients with grade B, 37.4% (*N* = 43) had hyperammonemia; however it was not statistically significant (*z*-score = 0.0 and *P* > 0.05). The overall Chi-square test result showed a strong evidence of relationship between hyperammonemia and increasing severity of liver cirrhosis (Child-Pugh grade); *χ* ^2^(2) = 27.46 and *P* < 0.001. The effect size was statistically significant too; Phi = 0.40 and *P* < 0.001.

## 4. Discussion

Cirrhosis of the liver was the cause of 1.2 million deaths in 2013 in comparison with less than a million deaths in 1990 [11]. Chronic viral hepatitis is the main culprit behind liver cirrhosis. HCV alone infects more males than females in the fifth decade of life and is more common in drug abusers and blood transfusion dependent patients [12–15]. Our results are comparable to these statistics.

Hyperammonemia is associated with both hepatic encephalopathy and fetor hepaticus [16]. Similarly, Iwasa et al. did a study to clarify the relationships amongst psychometric testing results, serum ammonia (NH3) levels, electrolyte disturbance, and degree of inflammation and their correlations with the development of hepatic encephalopathy (HE). They concluded that serum ammonia level was significantly higher in patients with hepatic coma [17]. Our results are comparable with these observations.

As gut microbiota is important in producing ammonia, their eradication with certain nonabsorbable antibiotics like rifaximin is the mainstay of treatment of hepatic coma. Studies suggest that the beneficial effects of both lactulose and rifaximin could be due to a change in the microbial metabolic function, leading to bacterial survival disadvantage as well as an improvement in dysbiosis via direct eradication [18, 19]. This data is supported further by our study, as most of the patients showed dramatic response to treatment with rifaximin and lactulose.

It is notable that 15% of the patients in our study group were cachectic. This is in contrast with other studies where muscle wasting was seen in as much as 40–50% of the cirrhotic patients. This needs to be treated with regular exercises and nutritional support [20, 21]. As ammonia inhalation has been shown to cause vasodilation, it can be assumed that the arteriovenous shunting in hepatopulmonary syndrome or even the causation of esophageal varices is partly contributed by hyperammonemia [22, 23]. Our study results support these finding, as more than two-thirds of the patients in our study group had both hyperammonemia and accompanying esophageal varices.

Currently, hyperammonemia in cirrhotic patients is treated with laxatives (lactulose), antibiotics like rifaximin, and/or neomycin and the administration of branched chain amino acids (BCAAs) and so forth. However, in an experimental study by Kosenko et al., the erythrocytes obtained from mice were loaded with the enzyme glutamine synthetase, called ammocytes and infused into hyperammonemic mice. The results were exceptional, as up to 50% reduction in ammonia levels of hyperammonemic mice was seen. The researchers observed that ammocytes were able to maintain their integrity, normal energy metabolism, and the inserted glutamine synthetase activity [24].

The results of our studies were according to expectations of the authors, but we recommend further studies, including bigger sample sizes, to clarify the true link between ammonia and hepatic encephalopathy and severity of cirrhosis.

## 5. Conclusion

It can be concluded that both chronic hepatitis B and chronic hepatitis C are the leading causes of liver cirrhosis in our region. Furthermore, ammonia in cirrhosis is positively related with both hepatic encephalopathy and Child-Pugh grade.

## Figures and Tables

**Table 1:** Details of different causes of chronic liver disease in our study group.

Etiology of CLD	Number of patients	Patients (%)
HCV	73	42.6%
HBV	41	24%
Both HCV & HBV	8	4.7%
Metabolic (Wilson's disease, etc.)	9	5.3%
Autoimmune (PBC, PSC, AIH, etc.)	11	6.4%
NAFLD	21	12.3%
Alcoholic liver disease	3	1.8%
Idiopathic etiology & others	5	2.9%

**Table 2:** Division of the patients in different groups as per Child-Pugh scoring system.

Child-Pugh grade	Number of the patients	% of the patients
A	47	27.5%
B	64	37.4%
C	60	35.1%

**Table 3:** Number of patients who had developed different complications of chronic liver disease.

Feature	Number (*N*)	Percent (%)
Hepatopulmonary syndrome (HPS)	28	16.4%
Hepatorenal syndrome (HRS)	43	25.1
Spontaneous bacterial peritonitis (SBP)	112	65.5
Esophageal varices	117	65%
Hypoglycemia	20	12%
Hepatocellular carcinoma (HCC)	24	14%

**Table 4:** Number of patients having different detectable liver abnormalities on an abdominal ultrasound.

Ultrasonography findings	Number (*N*)	Percent (%)
Coarse liver	171	100%
Irregular liver margins	154	90.1%
Dilated portal vein (PV)	151	88.3%

**Table 5:** Distribution of patients into different grades of hepatic encephalopathy (HE) as per West-Haven criteria.

Grade of HE	Number (*N*)	Percent (%)
No encephalopathy	23	13.5%
Grade 1 HE	41	34%
Grade 2 HE	38	22.2%
Grade 3 HE	40	23.4%
Grade 4 HE	29	17%

**Table 6:** Chi-square test statistics of distribution of hyperammonemia across different grades of hepatic encephalopathy (HE).

HE Grade	Patients (%) with hyperammonemia	*z*-score
No HE	1.7%	−6.4
Grade 1	15.7	−3.7
Grade 2	22.6	0.2
Grade 3	34.8	5
Grade 4	25.2	4.1
*χ* ^2^ value	*χ* ^2^(4) = 79.58, *P* < 0.001, Phi = 0.68, and *P* < 0.001	
